# Contrary to Expectations: Does Context Influence the Processing Cost Associated with Negation?

**DOI:** 10.1007/s10936-021-09799-8

**Published:** 2021-07-27

**Authors:** Elena Albu, Oksana Tsaregorodtseva, Barbara Kaup

**Affiliations:** Department of Psychology, University of Tübingen, Schleichstraße 4, 72076 Tübingen, Germany

**Keywords:** Negation processing, Plausible denial, Minimal and extended contexts, Discourse markers

## Abstract

Negative sentences are hard to process when they are presented out of context. When embedded in a context of plausible denial their processing difficulty decreases or is completely eliminated. We investigated in six behavioral experiments whether the processing of negation is eased in a denial context triggered by discourse markers (e.g. *Contrary to expectations, John has/hasn’t eaten the soup*). In order to investigate the necessary conditions for a context of plausible denial to reduce the processing cost of negation, we contrasted the processing of affirmative and negative sentences in minimal and extended denial and non-denial contexts (represented by either no context or a different type of context). We expected significantly longer response times (RTs) for negative sentences in comparison with affirmation in non-denial contexts and similar RTs for affirmative and negative sentences in denial contexts. The results from a sensibility judgement task (Experiment 1 and 2) and from a self-paced reading paradigm (Experiment 3 and 4) showed two robust main effects of context and polarity but no significant interaction between the two factors, suggesting that the processing of negative sentences was not facilitated in a context of minimal denial triggered solely by discourse markers. However, when the discourse markers were replaced with the explicit mention of the expectation to be denied and longer narratives used, the processing difficulty was eliminated specifically in the denial contexts (Experiment 5). Furthermore, when the discourse markers were used in longer narratives, a facilitation effect was also found (Experiment 6). All in all, the present findings suggest that, although negative sentences are felicitous in a context of plausible denial, the interplay of pragmatic factors like relevance or informativity is decisive in easing their processing difficulty.

## Introduction

Negation is known to be more difficult to process, leading to more errors and longer reading and reaction times in comparison with affirmative sentences (Carpenter & Just, 1975; Clark & Chase, 1972; Fischler et al., 1983). The observed processing difficulty is correlated with the presentation of negative sentences out of context. However, when negation is embedded in a legitimizing context, its processing cost decreases and is even eliminated in some cases (for an overview, see Kaup & Dudschig, 2020). One such context is the context of plausible denial, by means of which the property to be denied is previously activated either explicitly or implicitly (Glenberg et al., 1999; Lüdtke & Kaup, 2006; Orenes et al., 2016; Wason, 1965). The processing difficulty is also eased when negation addresses a negative QUD, which can be underlined by grammatical structures like cleft sentences (Tian et al., 2010, 2016). Other pragmatic factors like informativity and polar QUDs are also correlated with the processing ease of negative sentences (Nordmeyer & Frank, 2014; Xiang et al., 2020).

The property being denied can be expressed via multiple modalities, namely it can be embedded in a verbal context by means of oral or written sentences, or in a non-verbal context by using pictures. Although there is evidence supporting the claim that the context of plausible denial leads to a facilitation in the processing of negative sentences, it is not entirely clear which factors are directly responsible for this effect. The current study aimed at providing more information concerning this issue. To that end, we contrasted the processing of affirmative and negative sentences in minimal and extended verbal contexts expressed by means of written sentences. In what follows, we will briefly present how the context of plausible denial was discussed in the literature and afterwards introduce our study.

Starting from the assumption that the function of negative statements is generally to emphasize that a fact was contrary to an expectation, Wason (1965) tested two hypotheses which differed in the way in which the negated property was coded. The exceptionality hypothesis emphasized an exceptional item and a residual class (*Exactly one circle/seven circles is/are (not) …*). The ratio hypothesis highlighted a smaller and a larger class, the dissimilar item being negated in terms of a property which would have been initially affirmed of a discrete class (*Circle no. 7 is (not) ….*). Participants were faster when the negative sentence described the exception item in the exceptionality condition than when they described a smaller or a larger class in the ratio condition. It was concluded that negation was not inherently more difficult to process than affirmation when presented in a legitimizing context such as the context of plausible denial, as it allowed participants to accommodate either false propositions or the expectations that were denied as necessary steps in the comprehension of negative sentences.

Assuming that the processing difficulty associated with negative sentences is directly related to their presentation out of context, Glenberg et al. (1999) investigated how negative sentences are processed when presented in linguistic contexts governed by pragmatic constraints. Based on the premise that negation is used to address or to counter presuppositions held by listeners, participants were presented with longer narratives in which the target sentence (*The couch was (not) black*) was embedded in either a supportive context, where the relevant dimension was mentioned (*She wasn’t sure if a darkly colored couch would look the best or a lighter one*) or in a non-supportive context, where another dimension was mentioned (*She wasn’t sure what kind of material she wanted the couch to be made of*). In order to make the stories sound natural, additional introductory, intermediate and final sentences were used. The results showed that the negated sentences in the supportive context were processed as easily as affirmative sentences whereas the negated sentences in the non-supportive context were more difficult to process in comparison with the corresponding affirmative sentences. Glenberg et al. highlighted the importance of context in the processing of negative sentences and suggested that mentioning the relevant property dimension in the prior context led to the elimination of the processing cost associated with negative sentences.

Lüdtke and Kaup (2006) explored other features that rendered a context adequate for the processing of negative sentences. Concretely, they investigated whether the processing of negation is facilitated in a context in which the negated proposition was either explicitly mentioned as a potential possibility in the prior context or could be inferred as a plausible possibility in the given context. Participants read longer narrative stories in which a specific attribute of a particular entity which was previously introduced (*Danielle wondered whether the water would be warm/whether the water would be warm or cold/what the water would be like*) was either affirmed or denied (*The water was (not) warm*). The results showed that the processing of negative sentences, if at all, was facilitated when the proposition to be denied was explicitly mentioned in comparison to the case when no particular attribute was mentioned (*wondered what the water would be like*). However, mentioning one (*whether the water would be warm*) or two attributes of the entity (*whether the water would be warm or cold*) did not appear to be a decisive factor.

The authors further investigated the case when the proposition to be denied was presented in the form of an inference that could be drawn from the given context. Following a similar design, participants read narrative stories in which the inference (e.g. that the active/shy boy’s T-shirt would be clean/dirty) was denied by means of an affirmative (*The T-shirt was clean*) or a negative sentence (*The T-shirt was not dirty*). A facilitation effect was found when the context strongly implicated the negated proposition. These results corroborated the importance of presenting negative sentences in an appropriate context and provided evidence that the activation of the proposition to be denied could be inferentially triggered in addition to being explicitly mentioned.

In the same vein with previous studies, Nieuwland and Kuperberg (2008) investigated the impact context and world knowledge have on the processing of negative sentences in an ERP study. Accordingly, they contrasted the processing of negative sentences in pragmatically licensed and unlicensed contexts. Negation was considered pragmatically licensed when it was presented in a context in which it was used to reject something that plausibly may have been true (*With proper equipment, scuba-diving is very safe and often good fun*). In contrast, a pragmatically unlicensed negation (*Bulletproof vests are very safe and used worldwide for security*) was said to violate pragmatic principles such as informativity. In comparison with previous research, instead of using longer narrative stories, shorter expressions that highlighted world knowledge were used (e.g. *with proper equipment*). No additional processing cost was found in the case of pragmatically licensed negative statements. The processing difficulty that emerged when negation was pragmatically unlicensed was considered to be the consequence of violating pragmatic communicational principles.

Additional evidence supporting the claim that the processing of negative sentences is facilitated in a context where the negated situation was previously activated is provided by Orenes et al. (2016). In a visual world paradigm, the authors tested affirmative (*Her dad was rich*) and negative sentences (*Her dad was not poor*) in a neutral (*Her dad lived on the other side of town*), consistent (*She supposed her dad had enough savings*) and inconsistent context (*She supposed her dad had little savings*). The processing of negation was facilitated in the inconsistent context, namely the context in which the negated situation (‘her dad was poor’) was activated before participants were presented the negative sentence. However, in comparison with the results from behavioral studies, negative sentences were always slower to process than affirmation.

Overall, these studies present evidence from different paradigms that negative sentences are facilitated in a context of plausible denial and that the salience of the property to be denied, in the form of expectations, assumptions or previous statements, represents the decisive factor in triggering this effect. In contrast, when negative sentences are presented in the absence of a legitimizing context to guide towards their intended interpretation, they become more difficult to accommodate, warranting additional inferences and increasing their processing cost.

In the current study, we investigated the conditions under which the processing difficulty associated with negation can be reduced, rendering the processing of negative sentences comparable to the processing of affirmative sentences. There are more ways in which the situation to be denied can be activated and made accessible to both the speaker and the hearer in a verbal discourse: by being explicitly mentioned in a previous context, by contextual inference or by means of linguistic structures (Larrivée, 2012). In our study, the context of plausible denial is represented by linguistic contexts generated by discourse markers, such as *contrary to expectations* and synonym expressions like *unexpectedly*, *unpredictably*, *surprisingly* (in the following ‘discourse markers’). They render the affirmative and negative sentences felicitous by providing the context of plausible denial, namely by creating a contrast between an alternative state of affairs and the actual state of affairs. To illustrate, in the case of a sentence like *Contrary to expectations, John hasn’t eaten the soup,* the marker implies an alternative state of affairs derived from an expectation (‘It was expected that John would eat the soup’) which stands in contrast to the actual situation (*John hasn’t eaten the soup*) and raises the issue ‘whether John has eaten the soup’. On the contrary, when the negative sentence is presented out of context more questions are at issue (‘What has John eaten?’, ‘Who has eaten the soup?’, ‘Has John eaten the soup?’). This presumably leads to a delay in comprehending the sentence because people potentially compute more questions. Evidence supporting this claim is provided by visual world paradigm studies (Tian et al., 2016), where participants, when hearing *Bill has not opened his brother’s window*, look at both the negated (open window) and the actual state of affairs (closed window) for a rather long time (until hearing *window*) whereas when hearing *It was Bill who hasn’t opened his brother’s window* they look significantly faster at the actual state of affairs (closed window). By and large, the discourse markers (*contrary to expectations, surprisingly, unexpectedly, unpredictably*) grant a context of plausible denial for the upcoming sentences (*John has (not) eaten the soup*), namely by making the situation to be denied salient (It was expected that John would (not) eat the soup) and by clarifying what the question at issue is (whether John has eaten the soup).

In six behavioral experiments, we investigated whether a verbal context of plausible denial always leads to a facilitation of the processing cost associated with negation. In these experiments, we contrasted the processing of affirmative and negative sentences in contexts of plausible denial (in the following ‘denial context’) and in non-denial contexts. For the purposes of our argument, the denial context was represented either by solely discourse markers (Experiments 1 to 4) or by extended contexts, where more contextual information was provided (Experiments 5 and 6). In Experiments 1 and 2, participants responded to a sensibility-judgement task (with the judgement times serving as dependent variables) and in Experiments 3–6, they read the sentences fragment by fragment (with fragment reading times serving as dependent variables). We predicted an interaction between the factors *Context* and *Polarity* with (a) significantly longer response times (RTs) for negative compared to affirmative sentences in the non-denial contexts and (b) similar RTs for affirmative and negative sentences in the denial contexts. In Experiment 1, we compared the processing of affirmative and negative sentences in minimal denial contexts (*Contrary to expectations, John has/hasn’t eaten the soup*) with those in non-denial contexts (*John has/hasn‘t eaten the soup*) by means of a sensibility judgement task. To rule out that the results were due to the length disparity between the sentences in the two context conditions, in Experiment 2, we introduced expressions with an identical number of syllables in the non-denial context (*[Everybody is convinced that/ Everyone thinks that/ Based on what we know/ We believe that] John has/hasn’t eaten the soup*). This led to a change in the type of non-denial context from providing no context to providing a type of context which reports somebody else’s words. Furthermore, to ensure that the observed effects were not specific to the task employed, in Experiment 3 and 4, we replaced the sensibility judgement paradigm with a fragment-by-fragment self-paced reading task (*Contrary to expectations,//John has/hasn’t eaten the soup*). Following the same bipartite division of non-denial contexts (namely providing a reporting context or no context at all), in Experiment 3, we employed markers similar in length with those used in the denial context (*By all accounts,/Reportedly,/Apparently, Supposedly, John has/hasn’t eaten the soup*) while in Experiment 4, we did not offer any additional contextual information, similar to Experiment 1. The same pattern of results emerged in all four experiments, with two robust main effects of context and polarity but no significant interaction, indicating that the two factors were independent of each other. However, when the markers were replaced with the explicit mentioning of the expectation to be denied (*His parents expected John to eat the soup*) a facilitation effect of negation emerged in the denial contexts (Experiment 5), replicating the findings from previous research. A facilitation effect was also found when negative sentences were used in combination with the discourse markers in extended contexts, indicating that the facilitation effect was most likely triggered by the interplay between pragmatic factors like informativity or relevance (Experiment 6).

All in all, although the minimal contexts render the negative sentences felicitous, we could not find any evidence suggesting that these contexts eliminate the processing difficulty associated with negative sentences. Instead, embedding the sentences in longer narratives led to a facilitation effect, making the processing of negative sentences similar to the processing of affirmative sentences. This pattern of results suggests that although the context of plausible denial renders negation felicitous, the determining factors in triggering the facilitation effect are informativity or relevance.

## Experiment 1

### Participants

We recruited 135 participants who indicated English to be their native tongue from the Amazon Mechanical Turk platform (53 female, *M*_age_ = 35.68 years, *SD*_age_ = 10.31). The participation in the online experiment was voluntary and restricted to participants located in the United States. The participants were compensated with 2 US dollars and they all indicated English to be their native tongue.

### Stimuli

We used 40 sensical sentences with the following structure: *proper name* + *has/hasn’t* + *past participle* + *definite article* + *noun*. The experimental sentences were used either in the affirmative (J*ohn has eaten the soup*) or in the negative condition (*John hasn’t eaten the soup*). They were presented in two context conditions: denial contexts, where four discourse markers were added in initial position (*[Contrary to expectations,/Surprisingly,/Unexpectedly,/Unpredictably,] John has/hasn’t eaten the soup*) and non-denial contexts, where no additional information was provided (*John has/hasn’t eaten the soup*). Forty non-sensical filler sentences with an identical syntactic structure were also used in the four mentioned conditions (*[Contrary to expectations,/Surprisingly,/Unexpectedly,/Unpredictably,] Mark has/ hasn’t fed the shelves*)*.* The four discourse markers were equally distributed among the experimental and filler sentences, which were further distributed over four counterbalanced lists, so that one participant could only see one item in one of the four conditions (see Table 1 for all experimental conditions and "Appendix 1" for all items).

**Table 1:** Conditions Experiment 1 to 4

	Context	Affirmative	Negative
Exp. 1	Non-denial	*John has eaten the soup*	*John hasn’t eaten the soup*
	Denial	*Contrary to expectations, John has eaten the soup*	*Contrary to expectations, John hasn’t eaten the soup*
Exp. 2	Non-denial	*Everybody is convinced that John has eaten the soup*	*Everybody is convinced that John hasn’t eaten the soup*
	Denial	*Contrary to expectations, John has eaten the soup*	*Contrary to expectations, John hasn’t eaten the soup*
Exp. 3	Non-denial	*By all accounts, John has eaten the soup*	*By all accounts, John hasn’t eaten the soup*
	Denial	*Contrary to expectations, John has eaten the soup*	*Contrary to expectations, John hasn’t eaten the soup*
Exp. 4	Non-denial	*John has eaten the soup*	*John hasn’t eaten the soup*
	Denial	*Contrary to expectations, John has eaten the soup*	*Contrary to expectations, John hasn’t eaten the soup*

When choosing the experimental items, a pretest was conducted to make sure that the negative and affirmative sensical items did not differ significantly with regard to plausibility. To that end, we tested 60 sentences and 15 filler sentences which expressed different plausible events in a rating study. The pretest was implemented on the SoSciSurvey platform.^1^ We recruited 27 naive English speakers from the Amazon Mechanical Turk platform, who were excluded from the following experiments, and asked them to read and judge how plausible they found the situations described on a 7-point Likert scale, 1 standing for not plausible at all and 7 for very plausible. In the end, we selected 40 sentences which expressed very plausible situations and did not differ significantly in the affirmative and negative condition with regard to plausibility (*U* = 661.5, *p* = 0.182).

### Design and Procedure

The experiment took place online and it was run with English materials presented to English native speakers. The procedure was controlled by JsPsych (de Leeuw, 2015; Version 6.1.0). The experiment was accessible via a link and could be completed by participants using a common web browser.

The task of the participants was to read the sentences displayed on the screen and decide as quickly as possible whether these made sense by pressing the keys *f* and *j* on the keyboard, standing for *yes* and *no*, respectively. The experiment consisted of eight practice trials in order to let participants familiarize with the task, followed by 80 experimental trials and eight comprehension questions, which were presented after every tenth trial. Each experimental trial started with the presentation of a fixation cross briefly for 500 ms in the center of the screen. The sentences, written in black with a 16-font size, were displayed until participants pressed one of the two assigned keys on the keyboard. The key press allowed the next sentence to appear after a short period of 1000 ms. Response configurations were counterbalanced across participants. The trial order was pseudo-randomized by response and by condition so that trials of the same condition and requiring the same response would not appear more than twice in a row. The comprehension question trials followed the same procedure with one exception. After deciding whether the sentence made sense or not and pressing the corresponding key, participants were presented with a comprehension question regarding the information conveyed by the sentence and, additionally, the mention ‘if the answer is *yes/no*, please press *f/j*’ was displayed. The question could be answered by pressing the keys *f* and *j* on the keyboard, standing for *yes* and *no*, respectively. They were solely used to keep the participants focused on the task. The whole experiment took approximately 10–15 min to complete.

### Results

Based on an accuracy threshold of 80%,^2^ the set of participants was reduced to a final set of 79 participants (*M*_age_ = 38.38 years, *SD*_age_ = 11.34, 32 females). The data analysis only included the response times recorded for the experimental sentences (40 sensical sentences per participant). Error trials were deleted, which represented 5.47% of the relevant data. Based on visual inspection of the data plot, answers faster than 500 ms and slower that 6000 ms were considered absolute outliers and were also omitted (1.27% of the data). For further outlier elimination, we took differences between the participants as well as between the items into account. More specifically, we first converted the RTs to *z* scores by participant and then converted these *z* scores to *z* scores per item and condition. Consequently, we eliminated all trials for which this *z*-score was above/below 2/-2, respectively (4.59% of the relevant data).

**Fig. 1:**
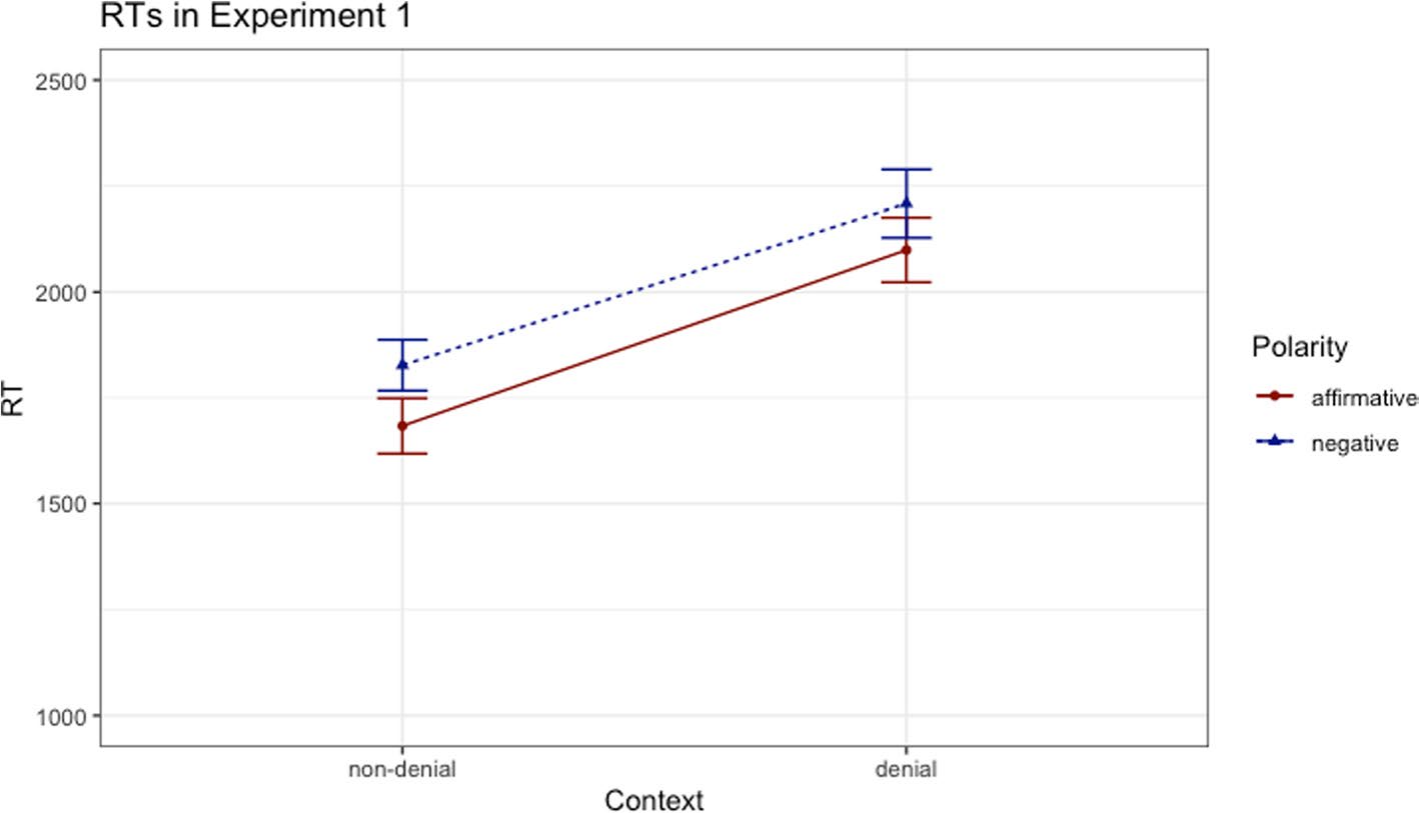
Response times (ms) in Experiment 1; error bars denote standard errors

The time required to read and evaluate the sensicality of the sentences served as dependent variable. Our design was a 2 × 2 factorial design with the factors *Context* (denial vs. non-denial) and *Polarity* (affirmative vs. negative). We analyzed the results by means of a linear mixed-effects model (LMEM).^3^ The analysis employed the free statistic software R (Version 4.0.3) and the R-package lme4 (Bates et al., 2015). The model included the factors *context* and *polarity* as fixed effects (dummy coded) and the random effects structure included by-participant and by-item intercepts. Other models with different complexity of random slopes did not converge. We created reduced models, dropping the fixed effects one by one and compared the base model with the reduced models using a likelihood ratio test. The base model significantly outperformed the reduced model without the fixed effect of *context* (*χ*^*2*^(1) = 248.86, *p* < 0.001, *β* = 119.99, *t* = -− 6.15), indicating that participants were faster to respond in non-denial contexts than in denial contexts. The base model also was better than the model without the fixed effect of *polarity* (*χ*^*2*^(1) = 24.39, *p* < 0.001 *β* = -− 91.82, *t* = 4.95), suggesting that participants responded faster to affirmative than to negative sentences, as shown in Fig. 1. Adding the interaction between *polarity* and *context* to the base model did not improve the model (*p* = 0.54).

This pattern of results indicated that the RTs in the denial and non-denial context conditions were not differently influenced by the polarity of the sentences. In other words, the denial context did not facilitate the processing of negative sentences, invalidating, therefore, our second prediction. Furthermore, the processing of both affirmative and negative sentences took longer in the denial contexts in comparison with the non-denial context. This aspect may be the consequence of the length difference between the sentences in the two context conditions: whereas the RTs included both the markers and the sentences in the denial context (e.g., ‘Contrary to expectations, John hasn’t eaten the soup’), in the non-denial context the RTs only reflected the sentences (‘John hasn’t eaten the soup’). Experiment 2 addressed this aspect and investigated the effect of context without the length confound. To that end, expressions with an identical number of syllables were added to the sentences in the non-denial context.

## Experiment 2

The analysis in Experiment 1 raised the concern that the observed pattern of results may have been influenced by the fact that the sentences in the denial and non-denial context conditions had different lengths. To remedy this, in Experiment 2 we introduced expressions with the same number of syllables in the non-denial context condition (*Everybody is convinced that/Everyone thinks that/ Based on what we know/We believe that*). This manipulation led to a change in the non-denial context, namely from providing no additional context to providing a different type of context by means of which somebody else’s words were reported.

### Participants

One hundred thirty-six participants were recruited from the Amazon Mechanical Turk platform (*M*_age_ = 36.95 years, *SD*_age_ = 10.73, 45 females). The participation in the online experiment was voluntary and restricted to the participants located in the United States and not having participated in the pre-test and Experiment 1. They were compensated with 2 US dollars. Three participants were excluded from the analysis as they didn’t indicate English as their mother tongue, reducing the initial set to 133 participants (*M*_age_ = 36.8 years, *SD*_age_ = 10.66, 45 females).

### Stimuli

We used the same experimental items and the same markers in the denial condition as in Experiment 1. In the non-denial condition, we added four expressions with the same number of syllables (*[Everybody is convinced that/Everyone thinks that/Based on what we know/We believe that] John has/hasn’t eaten the soup*). The same changes were applied to the filler sentences (*[Everybody is convinced that/Everyone thinks that/ Based on what we know/We believe that] Mark has/hasn’t fed the shelves)*. The addition of the expressions modified the type of the non-denial context, namely from providing no context at all to a context which was meant to report somebody else’s words. Although more contextual information was added, it nevertheless created a contrast with the denial context condition. As in Experiment 1, the denial context condition makes the situation to be denied salient but the non-denial context condition does not.

### Design and Procedure

The design, predictions and procedure were identical to those in Experiment 1. The experiment lasted 15 min on average.

### Results

Based on the same accuracy threshold of 80% as in Experiment 1, the set of 133 participants was reduced to a final set of 62 participants (*M*_age_ = 39.6 years, *SD*_age_ = 11.13, 26 females). The fillers were excluded from the data analysis as well as the error trials (5.4% of the relevant trials). As in Experiment 1, we removed absolute outliers (more than 6000 ms and less than 500 ms; 2.38% of the relevant data) as well as relative outliers, employing the same procedure as before (4.96% of the relevant data).

**Fig. 2:**
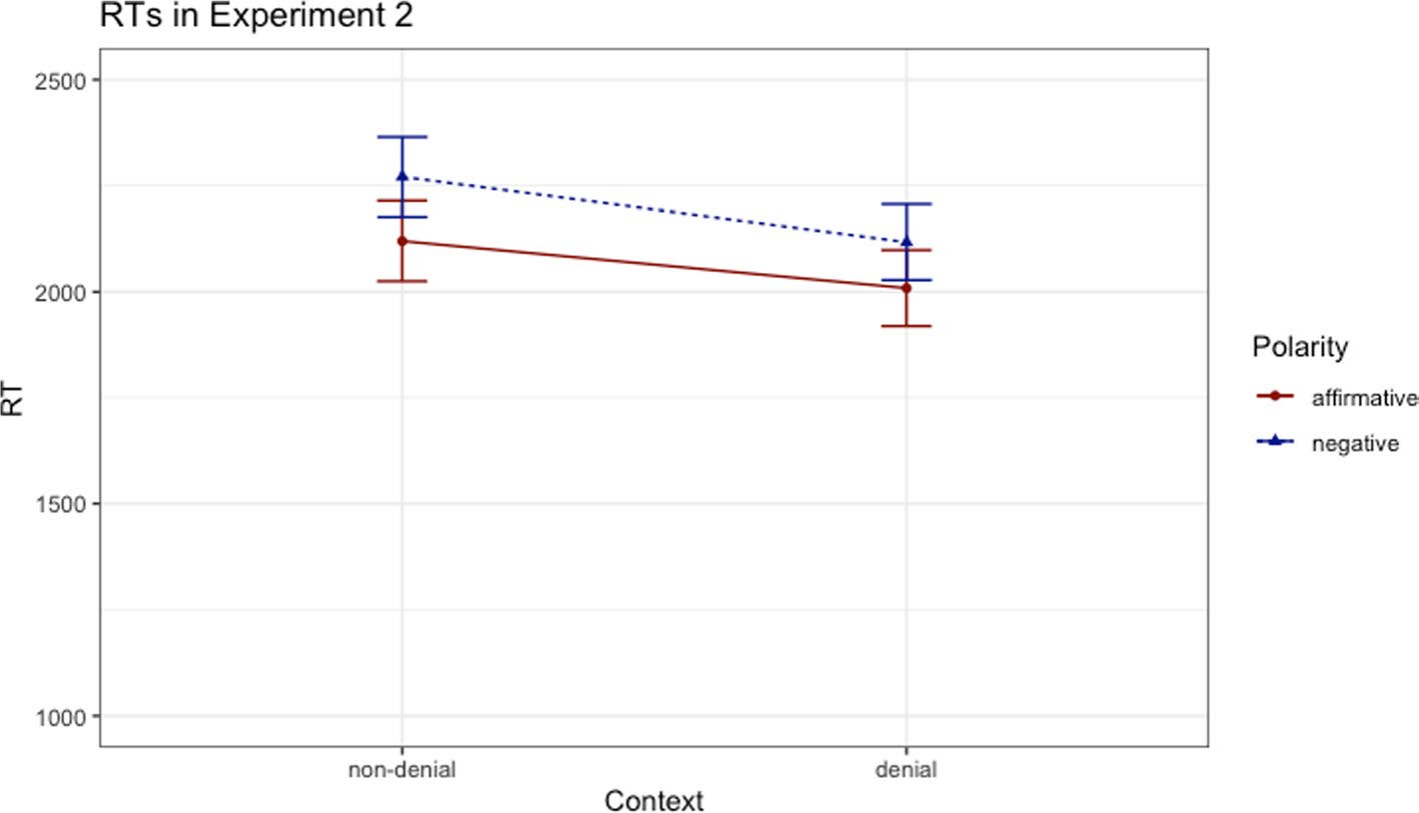
Response times (ms) in Experiment 2; error bars denote standard errors

The same statistical procedure was used, namely a comparison between a linear mixed effects model^4^ including fixed effects of *polarity* and *context* (dummy coded) with models without one of the two mentioned fixed effects. Like in Experiment 1, the base model significantly outperformed the model without the polarity factor (*χ*^*2*^(1) = 35.22, *p* < 0.001, *β* = 139, *t* = 5.96), indicating a main effect of polarity. The base model also significantly outperformed the model without the context factor (*χ*^*2*^(1) = 33.32, *p* < 0.001, *β* = -− 36.71, *t* = -− 5.8), indicating a main effect of context. The comparison between the base model without interaction with the model with interaction showed that the interaction between the relevant factors was not significant (*p* = 0.155). The results are depicted in Fig. 2.

All in all, the analysis revealed that the factors context and polarity did not influence each other, the overall pattern of effects replicating the findings in Experiment 1. In contrast with Experiment 1, there were longer RTs in the non-denial context condition, possibly reflecting the complexity of the grammatical structures employed. Although the expression in the non-denial context condition had the same number of syllables as the markers used in the denial context condition, the grammatical structure of the former involved two sentences in which more agents and more actions were involved. In sum, Experiment 2 adds to the evidence that the processing of negation was facilitated neither in a context of plausible denial nor in a context in which somebody else’s words were reported. One caveat of Experiment 1 and 2 is that the RTs in the sensibility judgement task included the time required for response decision and preparation and, therefore, might not be ideal to capture differences in processing times. Consequently, in Experiments 3 and 4 we used a fragment-by-fragment self-paced reading paradigm which has the advantage of being a purer measure of processing time.

## Experiment 3

In Experiment 3, we replaced the sensibility-judgement task with a self-paced reading task. Participants were asked to read affirmative and negative sentences fragment-by-fragment (*Contrary to expectations,//John has/hasn’t eaten the soup*). We did not opt for a word-by-word self-paced-reading task in the attempt to ensure that negation was integrated into the sentence meaning, on the one hand, and that the scope of negation was not too ambiguous to comprehend negation, on the other hand. In other words, we wanted to make sure the participants look at the event expressed as a whole (which is in line with the QUDs of the sentences) and not put focus on separate sentence components. Furthermore, there were longer RTs in the non-denial context condition in Experiment 2, in contrast with Experiment 1, where there were longer RTs in the denial context condition. We assume that this difference in RTs was due to the potential grammatical complexity of the expressions used in Experiment 2. To eliminate this additional complexity in the non-denial context condition, we used this time affirmative and negative sentences in combination with discourse markers in both the denial and non-denial context conditions while keeping the relevant difference between the conditions (namely making the situation to be denied salient in the denial context condition) intact.

### Participants

We recruited 167 volunteers (*M*_age_ = 39.34 years, *SD*_age_ = 13.59, 55 females) from the Amazon Mechanical Turk platform. The participation in the experiment was restricted to participants located in the United States and not having completed the pretest and the previous two experiments. Participants received 2 US dollars for carrying out the experiment. Five participants were excluded from the analysis as they did not indicate English as their mother tongue, reducing the initial set to 162 participants (*M*_age_ = 38.65 years, S*D*_age_ = 12.02, 55 females).

### Stimuli

The experimental items consisted of the same 40 target sentences used in the first two experiments. The same markers were used in the denial context condition whereas the expressions in the non-denial context used in Experiment 2 were replaced with markers having the same reporting function (*by all accounts/reportedly/apparently/supposedly*) (see Table 1). We changed the fillers because they were nonsensical in Experiments 1 and 2 due to the task. In this experiment we employed 120 sensical fillers with different grammatical structures in order to conceal the target sentences and to avoid participants losing interest and, therefore, not reading the sentences carefully (see Table 2 for illustrations). Eighty fillers were in the present tense (*Sharks are fish*), passive voice (*The contract will be signed tomorrow*), the -ing aspectual form (*The weather is getting colder*) and using modal verbs (*Carol should have departed by now*) and 40 fillers had a similar syntactic structure as the experimental items. This led to an equal distribution of sentences employing present perfect and other grammatical structures. In addition to the discourse markers used in denial and non-denial contexts, we also used eight additional markers (*in summary, in addition, as a result, lastly, consequently, in any case, overall, nevertheless*), leading to a balanced distribution of the markers as well among the experimental and filler sentences.

**Table 2:** Experimental and fillers sentences Experiment 1 to 4

	Context	Experimental sentences	Fillers
Exp. 1	Denial	*Contrary to expectations, John has/hasn’t eaten the soup*	*Contrary to expectations, Mark has/hasn’t fed the shelves*
	Non-denial	*John has/hasn’t eaten the soup*	*Mark has/hasn’t fed the shelves*
Exp. 2	Denial	*Contrary to expectations, John has/hasn’t eaten the soup*	*Contrary to expectations, Mark has/hasn’t fed the shelves*
	Non-denial	*Everybody is convinced that John has/hasn’t eaten the soup*	*Everybody is convinced that Mark has/hasn’t fed the shelves*
Exp. 3	Denial	*Contrary to expectations, John has/hasn’t eaten the soup*	*Contrary to expectations, Mark has/hasn’t fed the shelves*
	Non-denial	*By all accounts, John has/hasn’t eaten the soup*	*Reportedly, Pam has/hasn’t washed the carpet*
Exp. 4	Denial	*Contrary to expectations, John has/hasn’t eaten the soup*	*Contrary to expectations, Max has/hasn’t seen that movie before*
	Non-denial	*John has/hasn’t eaten the soup*	*Pam has/hasn’t washed the carpet*

### Design and Procedure

The experiment took place online and the procedure was controlled by *JsPsych* (de Leeuw, 2015). It consisted of four practice trials, followed by 160 experimental trials and 24 comprehension statements. Each trial started with the presentation of a centered fixation cross for 500 ms, followed by the two fragments, the marker and the main sentence. To go from one fragment to the next, participants pressed the space bar on the keyboard. Both fragments were written in black and used a 16-font size. After the second fragment, a blank screen appeared for 1000 ms and a new trial started afterwards. The order in which the items appeared was randomized for each participant. The participants’ task was to carefully read the sentence fragments and to evaluate the statements that were displayed approximately after every seventh sentence. Two answer possibilities, ‘correct’ and ‘incorrect’, were presented as clickable buttons on the screen. The left vs. right position of the buttons on the screen was randomized. Accuracy on the comprehension statements served as a way to verify that participants were paying attention to the task. The design and predictions were identical to those in the previous experiments. The experiment lasted 15 to 20 min to complete.

### Results

Based on a threshold of 80% accuracy on the comprehension statements, the set of participants was reduced to a final set of 61 participants (*M*_age_ = 39.73 years, *SD*_age_ = 13.09, 23 females). We again removed absolute outliers (RTs faster than 500 ms and slower than 5000 ms; 8.81% of the data) as well as relative outliers, employing the same procedure as before (3.52% of the data).

**Fig. 3:**
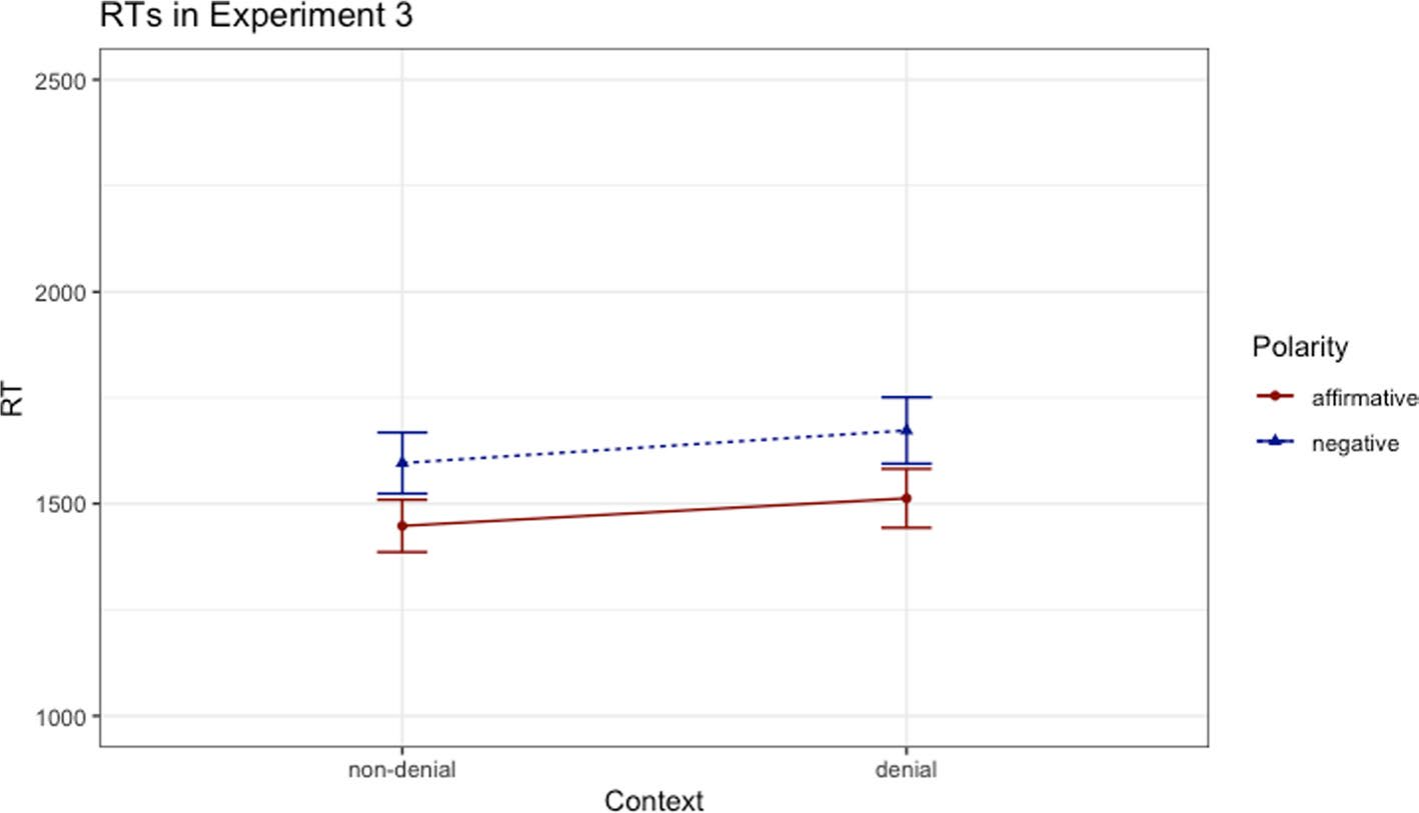
Reading times (ms) in Experiment 3; error bars denote standard errors

Like in the previous experiments, we analyzed the data by means of a linear mixed-effects model.^5^ The RTs measured for the fragment representing the sentences without the markers (*John has/hasn’t eaten the soup*) served as the dependent variable for our analyses. Similar to the previous experiments, our base model used to predict RTs included fixed effects for polarity and context (dummy coding) as well as by-participant and by-item random intercepts. We then employed the same procedure as described previously, comparing the base model with the reduced ones. The base model was a better fit for the data in comparison with the model without polarity as a fixed effect (*χ*^*2*^(1) = 68.36, *p* < 0.001, *β* = 165.55, *t* = 8.34) and a better fit than the model without context as a fixed effect (*χ*^*2*^(1) = 13.77, *p* < 0.001, *β* = 74.7, *t* = 3.72). Overall, the results thus revealed a main effect of polarity, indicating that participants read faster the affirmative than the negative sentences, and a main effect of context, showing that participants were faster to read the sentences in the non-denial context condition than in denial context condition, as shown in Fig. 3. Once again, the model with interaction did not improve the model without interaction (*p* = 0.78). It is worth noticing that participants took longer to read the sentences in the denial context condition, the pattern being similar to the one observed in Experiment 1. Thus, it seems that we were probably right to attribute the processing difficulty observed in the non-denial context condition in Experiment 2 to the grammatical complexity of the linguistic expressions used.

All in all, the finding that there was no interaction of the two factors replicated the findings in Experiment 1 and 2, suggesting that the processing of negative sentences does not appear to be facilitated in a context of plausible denial triggered by discourse markers. If Experiment 3 was meant to directly replicate the findings in Experiment 2 by comparing the same type of non-denial context, Experiment 4 attempted to replicate the findings of Experiment 1, when no additional information was provided in the non-denial context condition.

## Experiment 4

Using a self-paced reading paradigm, Experiment 4 aimed at testing whether a facilitation effect could be observed in the processing of affirmative and negative sentences in the denial context condition (*Contrary to expectations,//John has/hasn’t eaten the soup*) in comparison with the situation when the sentences were not embedded in any kind of context (*John has/hasn’t eaten the soup*).

### Participants

We tested 81 participants (*M*_age_ = 33.21 years, *SD*_age_ = 15.68, 22 males), recruited from the Prolific platform. The participation was limited to volunteers located in the UK and the US. All participants received 4 pounds for their participation. We excluded four participants as they did not report English as being their native language, which reduced the set of participants to 77 (*M*_age_ = 37.77 years, *SD*_age_ = 12.47, 22 males).

### Stimuli

The set of experimental sentences was identical to the one in Experiment 1 (see Table 1). We again used 120 sensible filler sentences for the same reasons as in Experiment 3 (see Table 2). In contrast to the previous experiment, only 60 filler sentences were used in combination with discourse markers. In the same spirit as before, we varied the markers used with the filler sentences which led to an even overall distribution of sentences in the denial and non-denial context conditions, namely 80 sentences used with markers and 80 sentences without.

### Design and Procedure

In the previous experiments we experienced a significant data loss. In the attempt to prevent it, we enlarged the amount of comprehension statements to 48 trials, making them appear randomly after approximately every three sentences. Additionally, the comprehension questions were accompanied by a feedback message only when the answer was not correct ‘Your answer is wrong!’ and, consequently, participants had to wait for five seconds until the next sentence was displayed. The predictions were identical to those formulated in the previous experiments. The experiment lasted 15–20 min.

### Results

**Fig. 4:**
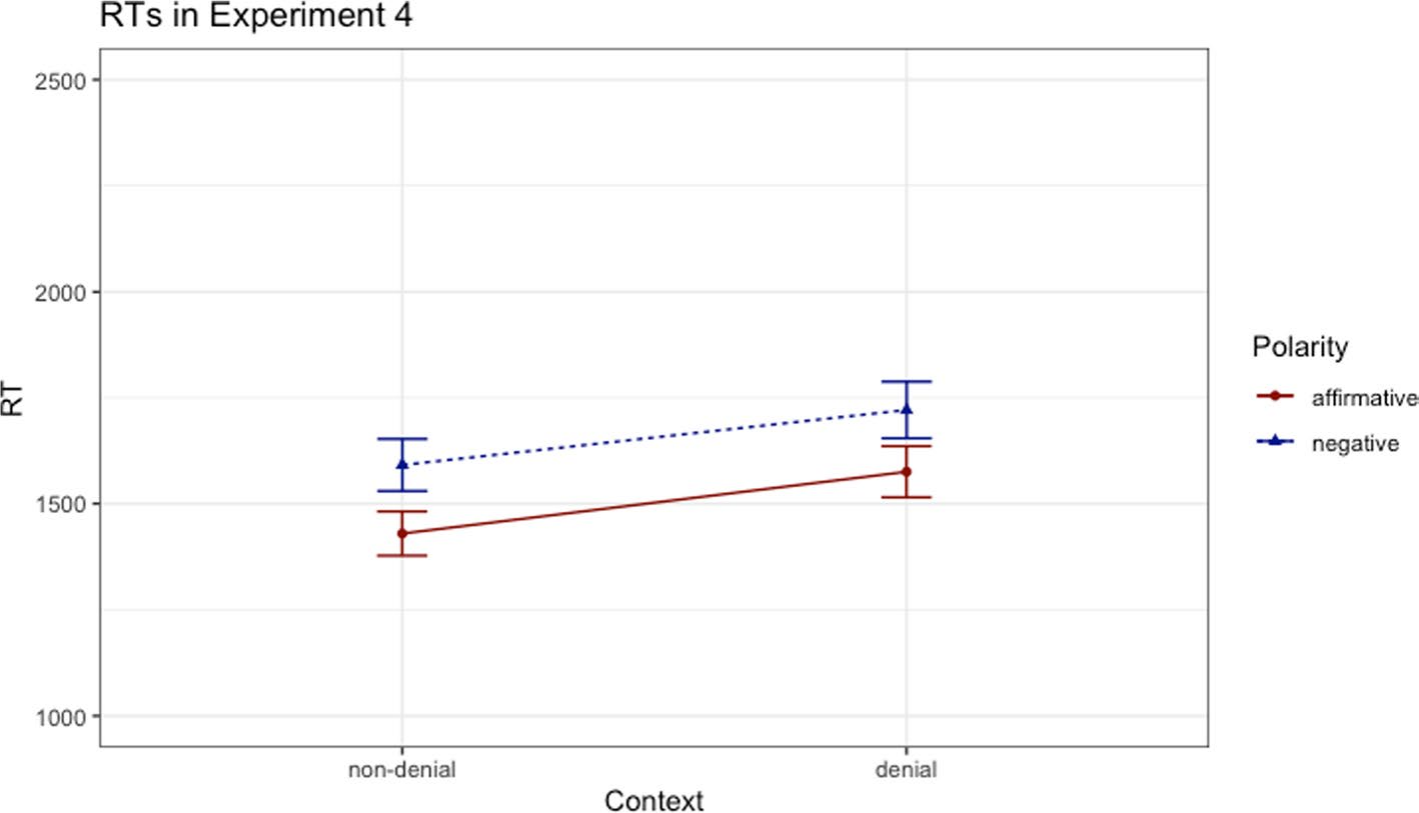
Reading times (ms) in Experiment 4; error bars denote standard errors

After setting the accuracy threshold of 80% on the comprehension statements, the data of a remaining set of 64 participants was analyzed (*M*_age_ = 31.72 years, *SD*_age_ = 12.16, 17 males). We followed the same procedure of excluding outliers as in the previous experiments: based on visual inspection we deleted absolute outliers (RTs faster than 500 ms and longer than 6000 ms; 7.66% of the data), and relative outliers were determined according to the same procedure as in the previous experiments (5.16% of the data). The relevant data was analyzed by means of a linear mixed-effects model,^6^ in which the RTs measured for the fragments representing only the sentences (*John has/hasn’t eaten the soup*) served as dependent variable. The model without any random slopes proved to be the most complex model that converged. Thus, as in our previous analyses, the base model in this analysis consisted of fixed effects for polarity and context (dummy coding) and by-participant and by-item random intercepts. The results revealed a main effect of polarity and a main effect of context, inasmuch as the base model outperformed the model without the fixed effect of polarity (*χ*^*2*^(1) = 38.64, *p* < 0.001, *β* = 165, *t* = 6.24) and the model without the fixed effect of context (*χ*^*2*^(1) = 29.24, *p* < 0.001, *β* = -− 43.79, *t* = -− .43). The model with the added interaction between the factors polarity and context did not again improve the model with the two main effects (*p* = 0.771), as shown in Fig. 4.

Overall, Experiment 4 replicated the previous pattern of results, indicating once more that the factors polarity and context do not influence each other. Participants were faster to read the affirmative sentences than the negative sentences and slower to read the sentences in the denial context condition than those in the non-denial context condition. All things considered, the findings in all four experiments converge, suggesting that the processing of negative sentences is not eased in a minimal context provided exclusively by discourse markers. Although these markers render the affirmative and negative sentences felicitous, they do not seem to satisfy the necessary conditions to decrease the processing cost associated with negation. In other words, making the property to be denied salient appears to be an insufficient condition and brings into question the factors responsible for the processing facilitation.

However, before committing to this conclusion, an alternative explanation may be offered for these findings. It was assumed that the discourse markers (*Contrary to expectations, John has/hasn’t eaten the soup*) imply an alternative state of affairs derived from a contextual expectation (‘It was expected that John would/wouldn’t eat the soup’). Concretely, they were assumed to raise a polar question at issue, targeting the entire event (‘Has John eaten the soup?’). Although no additional context is provided and no specific element is focused explicitly, it is possible that other questions at issue may have been activated (Who has eaten the soup? What has John eaten?), an aspect which could have contributed to the increased processing cost of the sentences in the denial context.

Building on Glenberg et al. (1999) and Lüdtke and Kaup (2006), in Experiment 5 we aimed at investigating whether negative sentences become easier to process in narrative stories where more contextual information was provided and the question at issue is clearly defined. To that end, we replaced the discourse markers with the explicit mention of the expectation which emphasized the alternative state of affairs and provided more contextual information in the form of short stories. It is possible that, in addition to being felicitous, negative sentences need to be informative and relevant.

## Experiment 5

Based on the assumption that the processing of negative sentences may be correlated with pragmatic factors like informativity (Nordmeyer & Frank, 2014) and relevance^7^ (Tian et al., 2016), Experiment 5 tested whether the explicit mention of the expectation focusing on the alternative state of affairs could lead to a decrease of the processing cost associated with negative sentences. Following the design used in Glenberg et al. (1999) and Lüdtke and Kaup (2006), we provided two additional sentences meant to set some background information and explicitly mentioned the alternative state of affairs in the denial context condition, whereas no alternative was mentioned in the non-denial context condition. If a facilitation effect is found in the denial context condition, then it can be attributed to pragmatic factors like informativity or relevance triggered in longer contexts.

### Participants

Eighty volunteers (*M*_age_ = 35.92 years, *SD*_age_ = 12.39, 29 males) participated in the experiment. They were recruited from the Prolific platform and their participation was restricted to participants located in the UK and in the US and not having taken part in Experiment 4. They were compensated with 4 pounds and all of them reported that they were English native speakers.

### Stimuli

We created 40 short stories that involved the same experimental items from the previous experiments. The stories commenced with two introductory sentences meant to set the necessary background information (*John enjoys having dinner with his parents but unfortunately he had to work late last week. His parents put the leftovers in the fridge*). Then the explicit mention of the expectation was introduced in the denial context condition (*They expected/ didn’t expect John to eat the soup*) while no specific expectation was created for the non-denial context condition (*They wondered what John would do*). In order to render the narration coherent, the target sentence (*John had/hadn’t eaten the soup*) was preceded by an introductory fragment (*The following morning his parents discovered that*). In accordance with grammatical rules, the present perfect aspectual form used in the previous experiments was replaced with the past perfect form. The story ended with a comprehension question which could target information from the entire given context. To add variety to the items, more expressions were used for the explicit mention of the expectation in the denial context condition (*expect, assume, suppose*), either in the active or in the passive voice. Similarly, three synonym expressions were used in the non-denial context condition (*wonder, not know, be curios*). No filler items were used as we did not want to make the experiment last too long. Also, as the experimental items employed in this experiment were longer narrative stories and thus much less schematic than the experimental items in the previous experiments, including filler items seemed less relevant.

The difference between our stories and those presented in Glenberg et al. (1999) and Lüdtke and Kaup (2006) is found in the way in which the expectation was introduced in the denial context: whereas in the previous studies the target sentence was expressed in the form of an indirect question (*She wasn’t sure if; She wondered whether*) and was followed by the mention of one or two characteristics of the relevant dimension, in our study the expectation was more assertive, bringing into foreground the alternative situation and raising the question *whether p* (*whether John has eaten the soup*) in both polarities. An illustration of the materials can be found in "Appendix 2".

### Design and Procedure

The experiment was conducted in English and took place online. The procedure was controlled by JsPsych (de Leeuw, 2015). Participants were asked to read all 40 stories and to answer the comprehension questions corresponding to each story. Each trial started with the presentation of a fixation cross for 500 ms, followed by the presentation of the sentences. The text presentation was either sentence by sentence or fragment by fragment (for the introductory fragment and target sentence), self-paced by the participant. After reading one sentence, participants pressed the space bar on the keyboard and the next sentence was displayed. In order to measure RTs solely for the target sentences, the introductory fragment and the target sentences were presented fragment by fragment (*The following morning his parents discovered that//John had/hadn’t eaten the soup*). To go from the introductory fragment to the target sentence they followed the same procedure. The order in which the stories were presented was randomized for each participant. To ensure that participants read all the sentences carefully, the comprehension statements from the previous experiments were replaced with comprehension questions which targeted different information provided in the extended contexts and not only in the target sentence. The comprehension questions could be answered by two clickable buttons displayed on the screen, standing for ‘yes’ and ‘no’. The left versus right positioning of the buttons on the screen was randomized. Our design was again a 2 × 2 factorial design with the factors *Context* (non-denial vs. denial) and *Polarity* (affirmative vs. negative). The 40 stories were distributed over four counterbalanced lists, so that one participant could see a story in only one condition. The experimental session lasted approximately 30 min.

### Results

After setting an accuracy threshold of 80% on the comprehension questions, the set of participants was reduced to 67 participants (*M*_age_ = 37.24 years, *SD*_age_ = 12.81, 20 males). We again eliminated absolute outliers (RTs above 5000 ms and below 500 ms; 5.30% of the data) as well as relative outliers according to the same procedure as in previous experiments (4.89% of the data).

**Fig. 5:**
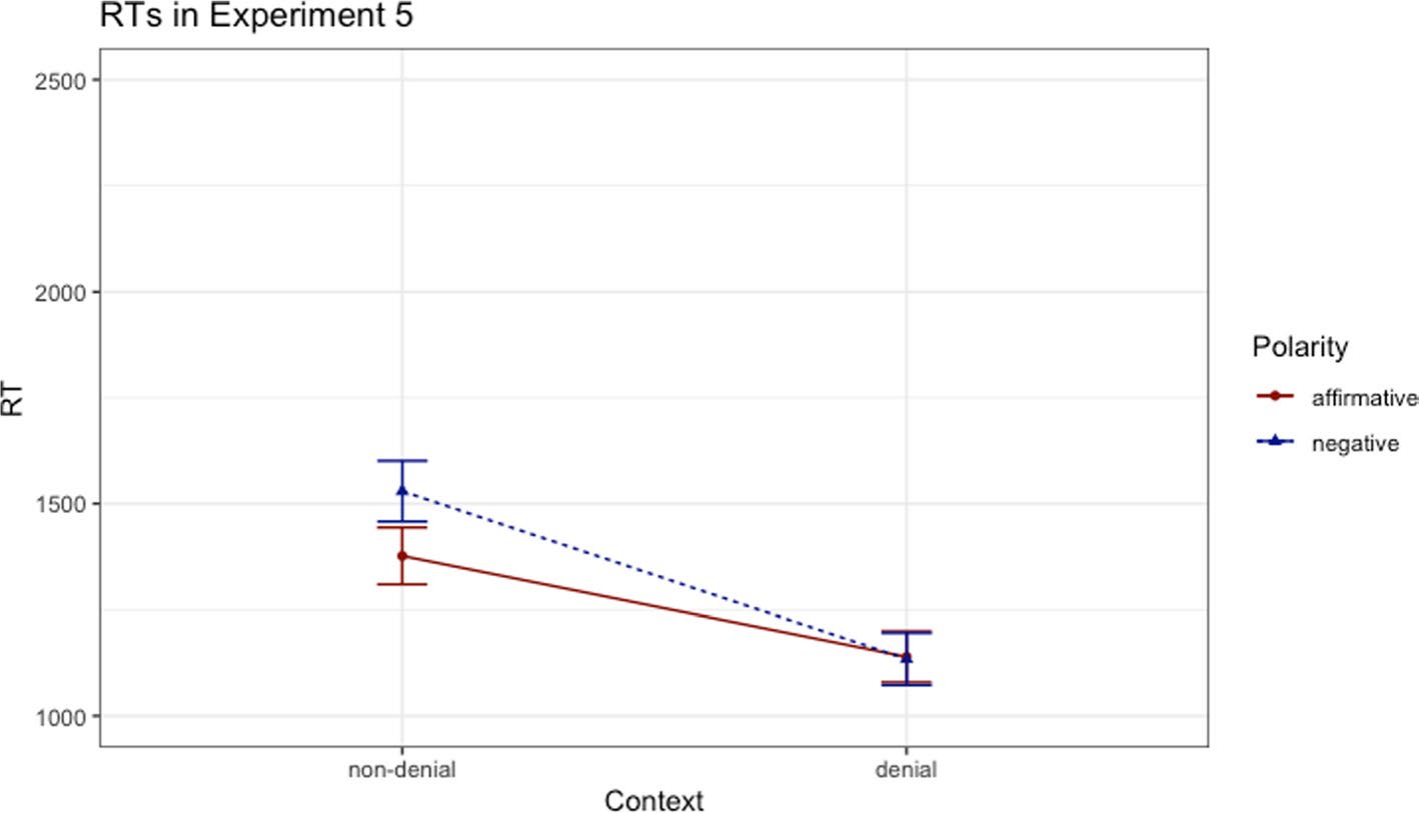
Reading times (ms) in Experiment 5; error bars denote standard errors

In order to examine the effect of context on the polarity effect we applied a linear mixed-effects model,^8^ as in the previous experiments. Once again, the model consisting of a fixed effect for both polarity and context (dummy coding) and random intercepts for participants and items was selected as the base model. The base model significantly outperformed the model without the fixed effect of polarity (*χ*^*2*^(1) = 16.58, *p* < 0.001, *β* = -− 5.07, *t* = -− 0.65). The base model also significantly outperformed the model without the fixed effect of context (*χ*^*2*^(1) = 352.44, *p* < 0.001, *β* = 238.41, *t* = 10.38). Most importantly, adding the interaction between the factors *polarity* and *context* to the base model led to an improvement of the model (*χ*^*2*^(1) = 24.06, *p* < 0.001, *β* = 159.51, *t* = 4.92), indicating that the processing difficulty of negative sentences differed between the non-denial and the denial context, as shown in Fig. 5. In contrast to the previous four experiments, a facilitation effect for the negative sentences was observed in the denial context condition in comparison with the non-denial context condition.

In sum, Experiment 5 presents evidence in favor of the claim that the processing difficulty associated with negative sentences is eliminated in a context of plausible denial operationalized by short stories, replicating the previous findings in Glenberg et al. (1999) and Lüdtke and Kaup (2006). The way in which the expectation is presented, introduced by means of an indirect question or used more assertively, does not seem to affect the pattern of results (because the same pattern was observed both in our and the previously reported studies). Overall, the findings in Experiment 5 cast new light on the factors that make a context of plausible denial effective. Whereas the results in our first four experiments showed that felicity is not sufficient to trigger a facilitation effect, the findings in Experiment 5 suggest that informativity or relevance may exert a powerful influence on the processing of negation. By and large, it is important to note that not every context of plausible denial leads to a facilitation effect.

However, the observed facilitation effect could also be accounted for in a different way. One can raise the concern that using the same words in the explicit mention of the expectation and in the target sentence in the denial context condition could influence the findings and lead to faster RTs in the denial than in the non-denial context condition, possibly leading to a floor effect in the denial context condition and thus not showing a polarity difference in this condition. Experiment 6 aimed at investigating whether the use of negative sentences in combination with discourse markers in extended contexts also leads to the observed facilitation effect without lexically priming the words in the target sentences. If this is the case, it can then be concluded that the effect is not (solely) triggered by lexical priming or predictability but rather by pragmatic factors and communicative principles.

## Experiment 6

In the attempt to draw a clear distinction between the influence of lexical priming and predictability of the same content words, on the one hand, and of pragmatic factors and communicative principles, on the other hand, Experiment 6 investigated whether negative sentences are facilitated when they are used in an extended context and the expectation is implicitly introduced by means of discourse markers. Concretely, our aim was to see whether a comparable facilitation effect for negative sentences can be also found when the target sentence (*John has/hasn’t eaten the soup*) is used in an extended context (*John enjoys having dinner with his parents but unfortunately he had to work late last week. His parents put the leftovers in the fridge*) and preceded by a bridging fragment which includes discourse markers (*The following morning his parents discovered that, contrary to expectations,/ surprisingly,/ unexpectedly,/ unpredictably,*). If this is the case, then the influence of lexical priming and predictability can be ruled out. Additionally, the current design allows us to check whether the discourse markers themselves pose comprehension difficulties, as it was speculated for the minimal contexts. If this is also not the case, then it can be concluded that the decisive elements in reducing the processing cost of negation are pragmatic factors like relevance or informativity, triggered by the extended contexts.

### Participants

Eighty-one volunteers (*M*_age_ = 36.03 years, *SD*_age_ = 13.13, 25 males) participated in the experiment for a compensation of 2.5 pounds. They were recruited from the Prolific platform and their participation was restricted to participants located in the UK and in the US and not having taken part in Experiment 4 and 5. All participants reported English to be their native tongue.

### Stimuli, Design and Procedure

We used the same 40 stories from Experiment 5 but we replaced the explicit mention of the expectation with the discourse markers. Therefore, in the denial context condition the first two introductory sentences (*John enjoys having dinner with his parents but unfortunately he had to work late last week. His parents put the leftovers in the fridge*) were followed directly by the linking fragment, which included the discourse markers (*The following morning his parents discovered that, contrary to expectations,*), and afterwards by the target sentence (*John has/hasn’t eaten the soup*). In contrast, in the non-denial condition, no discourse markers were employed (*John enjoys having dinner with his parents but unfortunately he had to work late last week. His parents put the leftovers in the fridge*. *The following morning his parents discovered that John has/hasn’t eaten the soup*). The same comprehension questions were used as in Experiment 5 (see "Appendix 3" for illustrations).

The design and procedure were similar to those in Experiment 5. The experiment lasted 15 min on average.

### Results

Based on threshold of 80% accuracy on the comprehension questions, the initial set of participants was reduced to 68 participants (*M*_age_ = 37.07 years, *SD*_age_ = 13.17, 19 males). Following the same procedure as in previous experiments, we eliminated absolute outliers (RTs above 5000 ms and below 500 ms; 2.10% of the data) as well as relative outliers according (4.44% of the data).

**Fig. 6:**
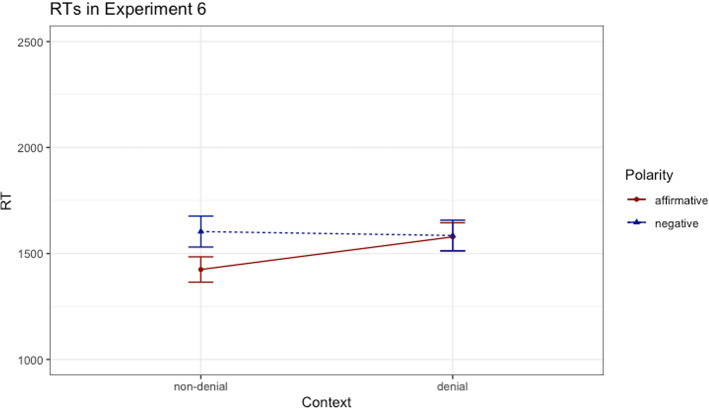
Reading times (ms) in Experiment 6; errors bars denote standard errors

The remaining data were analyzed by means of a linear mixed-effects model.^9^ Similar to previous experiments, the model consisting of fixed effects for both polarity and context (dummy coded) and random intercepts for participants and items was selected as the base model. The base model significantly outperformed the model without the fixed effect of polarity (*χ*^2^(1) = 25.68, *p* < 0.001, *β* = 159, *t* = 0.36) and the model without the fixed effect of context (*χ*^2^(1) = 16.24, *p* < 0.001, *β* = 9, *t* = -− 6.12), indicating a main effect of polarity and a main effect of context, respectively. Most importantly, and as in Experiment 5, adding the interaction between the factors polarity and context to the base model significantly outperformed the model without interaction (*χ*^2^(1) = 21.05, *p* < 0.001, *β* = 169, *t* = 4.60). In sum, the interaction between the two factors was statistically significant, indicating that the RTs were differently influenced by negation in the context conditions, as shown in Fig. 6.

Overall, an interaction between the two factors was found in both Experiment 5 and 6. There is, however, a difference between the findings in these two experiments: participants were faster to read the sentences in the denial context condition in Experiment 6, whereas reading times were faster in the non-denial condition in Experiment 6. The explicit mention of the expectation to be denied in the denial contexts of Experiment 5 probably led to a faster processing, which seems to be in line with the explanations concerning lexical priming and the predictability resulted from using the same content words. In contrast, the derivation of the implicit expectation necessary to compute the sentence meaning appears to be more costly in Experiment 6, explaining the difference between the results of the experiments. As a consequence of this difference, negative sentences were easier to process in the denial than in the non-denial context in Experiment 5, whereas in Experiment 6, negative sentences only indirectly gained from the denial context, in the sense that the more difficult denial context did not prolong the processing of negative sentences in the same way as it prolonged the processing of affirmative sentences. Overall, the pattern of results in Experiment 6 thus corroborates the view that the synergy between pragmatic factors like relevance or informativity, triggered by the additional contextual information provided, is the decisive factor generating the facilitation effect.

## General Discussion

Building on the assumption that negation is pragmatically felicitous when it is used to counter expectations and deny propositions, this study investigated under what conditions the processing difficulty associated with negative sentences is reduced in a context of plausible denial. In this regard, we compared the processing of affirmative and negative sentences in verbal contexts of plausible denial generated by discourse markers ([*Contrary to expectations,/Surprisingly,/Unexpectedly, Unpredictably,*] *John has/hasn’t eaten the soup*) in non-denial contexts, represented either by no context at all or by reporting contexts. The discourse markers render the affirmative and negative sentences felicitous by creating a contrast between an expected alternative state of affairs (*It was expected that John would/wouldn’t eat the soup*) and the actual state of affairs (*John has/hasn’t eaten the soup*). In other words, they make the expectation to be denied salient and raise the polar question at issue ‘Has John eaten the soup?’. In order to dissociate between the influence of different pragmatic factors like salience of the expectation to be denied, informativity or relevance, we carried out six behavioral experiments in which the processing difficulty was measured by the response and reading times of the target sentences. We employed minimal contexts, generated solely by discourse markers (Experiments 1 to 4), and short stories, where more contextual information was provided (Experiment 5 and 6). Our results showed that the processing difficulty associated with negative sentences is reduced when negative sentences are used in extended denial contexts but not in minimal denial contexts. In what follows, we will first offer some possible explanations for the findings in the minimal context and discuss what the potential implications are and, afterwards, expand on the results obtained in the extended contexts.

Although we used two paradigms, a sensibility judgement task (Experiment 1 and 2) and a self-paced reading (Experiment 3 and 4), and varied the type of the non-denial context (represented by either no context or a reporting context), the same pattern of results emerged for the minimal contexts with two robust main effects of *polarity* and *context* but no significant interaction. These findings indicated that the two factors were independent of each other, the RTs of the affirmative and negative sentences not being influenced by the context in which they were used. This pattern of results raises the speculation that the pragmatic felicity provided by the minimal context of plausible denial generated by discourse markers is not enough to ease the processing difficulty of negative sentences. In other words, making the expectation to be denied salient appears to be an insufficient condition in alleviating the processing difficulty of negation in a solely linguistic context. Furthermore, in Experiments 1, 3 and 4, the sentences in the denial context took longer to process than in the non-denial context, an aspect which also calls into doubt the assumption that the context of plausible denial unconditionally facilitates the processing of negation. One may argue that this aspect can be attributed to the varying linguistic complexity of the markers used. Although it is true that in Experiment 1 the RTs in the denial context also included the markers, this did not happen in Experiments 3 and 4, where the RTs exclusively reflected the target sentences in both context conditions. Furthermore, the denial context was contrasted with a reporting context in Experiment 3 and with no context at all in Experiment 4, and in both cases the denial context took longer to process. It is, therefore, more likely to attribute the processing difficulty to the comprehension of negative sentences in a minimal context of plausible denial.

In Experiments 5 and 6, we replaced the minimal contexts with short stories that provided more background information. In the attempt to replicate previous findings from the literature, in Experiment 5, we replaced the markers with the explicit mention of the expectation to be denied, whereas, in Experiment 6, we checked whether the discourse markers themselves pose comprehension difficulties or, alternatively, whether the processing difficulty was more likely caused by the insufficient pragmatic information provided in the minimal contexts. In contrast with the previous four experiments, the relevant interaction between context and polarity was statistically significant in both experiments, indicating that the two factors influenced each other. The results in Experiment 5 showed that negative sentences were facilitated in an extended context of plausible denial compared to an extended non-denial context. However, when interpreting the results of this experiment, it needs to be taken into account that the denial context used the same content words in the explicit mention of the expectation and in the target sentences and this might have also influenced the results. In order to dissociate between the influence of informativity or relevance, on the one hand, and lexical priming and predictability of repeating the content words, on the other hand, in Experiment 6, we used the same extended contexts from Experiment 5 but replaced the explicit mention of the expectation with the discourse markers. In contrast to Experiment 5, where the denial context indicated a clear facilitation of the processing difficulty in comparison with the non-denial context, the results in Experiment 6 showed that the derivation of the implicit expectation was more costly in the denial context. More importantly, however, the interaction between polarity and context was again significant in this experiment, ruling out an explanation solely based on lexical priming or predictability. Overall, these findings thus corroborate the view that negative sentences are felicitous in a context of plausible denial generated by discourse markers but that the processing difficulty often observed when looking at the processing of negative sentences seems to be directly correlated with pragmatic factors like informativity or relevance and is thus not eased in all contexts of plausible denial.

Another possible explanation for the differences in results in the minimal and extended contexts relates to the structure of the sentences. Although the markers are meant to render the sentences felicitous and set up the expectation to be denied, in the minimal contexts the readers could only identify what the expectation and the question at issue were once they have read the upcoming sentence. It is possible that the interpretation mechanisms, although predicted to alleviate the processing difficulty, may have been more costly than initially presumed. However, in Experiment 6, the interpretation of the target sentences accompanied by discourse markers did not seem to pose interpretation problems. Based on the current findings, we therefore do not find this explanation convincing. Instead, we presume that informativity or relevance were the factors responsible for reducing the processing difficulty of negative sentences. In Experiment 5 this was directly indicated by a particularly pronounced processing decrease for negative sentences in the denial context. In Experiment 6, however, this was more indirectly shown, namely by the fact that negative sentences showed a reduced increase in processing times in the denial vs the non-denial context compared to affirmative sentences. Additionally, as suggested by one of the reviewers, it is possible that there were also other factors involved in the narrative stories, like perspectivation or valence, but it is not completely clear at this point what their influence was. Future studies are necessary in order to provide more information on the different impact relevance and informativity may have, on the one hand, and highlight the influence of other pragmatic factors, on the other hand.

Building on Xiang et al., (2020), another aspect which calls for future investigation is the difference between informativity at a global level, namely the informativity of the sentence themselves, and informativity at the local level, namely whether the negative sentence is the most informative one to describe the state of affairs in the given context. The authors suggested that polar questions at issue render negative sentences informative at a global level (Xiang et al., 2020). Although all our sentences raised a polar question at issue, our findings seem to suggest that the informativity of the sentences alone in the minimal context could not reduce the processing difficulty. It is, of course, possible that this difference may be the result of comparing findings from solely linguistic contexts (our studies) and findings from visual contexts (Xiang et al., 2020). Future studies are necessary to broaden our understanding of the influence of the different types of contexts and levels of informativity on the processing of negative sentences.

## Appendix 1: *Items used in Experiment 1 to 4*

1. John has/hasn’t eaten the soup.
2. Emily has/hasn’t read the fable.
3. Caroline has/hasn’t tasted the cocktail.
4. Julien has/hasn’t deciphered the riddle.
5. David has/hasn’t spilt the coffee.
6. Jim has/hasn’t unloaded the containers.
7. John has/hasn’t ironed the shirt.
8. Aiden has/hasn’t washed the car.
9. Amy has/hasn’t finished the puzzle.
10. Ian has/hasn’t prepared the dinner.
11. Lucy has/hasn’t framed the photo.
12. Nick has/hasn’t decorated the Christmas tree.
13. Susan has/hasn’t mended the jeans.
14. Maria has/hasn’t sold the house.
15. Bob has/hasn’t chopped the log.
16. Eva has/hasn’t scratched the CD.
17. Jill has/hasn’t visited the library.
18. Lily has/hasn’t baked the pie.
19. Lucy has/hasn’t watered the plants.
20. Max has/hasn’t sent the postcards.
21. Sam has/hasn’t learnt the role.
22. James has/hasn’t lost the money.
23. Nick has/hasn’t climbed up the hill.
24. Taylor has/hasn’t the school.
25. Wilson has/hasn’t fired the manager.
26. Tom has/hasn’t planted the tobacco.
27. Meghan has/hasn’t interviewed the students.
28. Max has/hasn’t stolen the watch.
29. Barbara has/hasn’t the report.
30. Alex has/hasn’t underestimated the employees.
31. Helen has/hasn’t answered the question.
32. Sam has/hasn’t watched the film.
33. Harry has/hasn’t squeezed the oranges.
34. Laura has/hasn’t demolished the attic.
35. Robert has/hasn’t rejected their help.
36. David has/hasn’t bothered the driver.
37. Riley has/hasn’t collected the signatures.
38. Brian has/hasn’t cancelled the appointment.
39. Claudia has/hasn’t forged the signatures.
40. Ethan has/hasn’t the finish line.

## Appendix 2: ***Illustrations of the Items used in Experiment 5***

### Denial context

1. John enjoys having dinner with his parents but unfortunately he had to work late last week. His parents put the leftovers in the fridge. They expected/didn’t expect him to eat the soup. The following morning his parents discovered that John had/hadn’t eaten the soup.
2. Emily was invited to surprise birthday party where the guests were asked to recite different literary fragments. Most of them chose less known authors. Emily was/wasn’t supposed to read the fable. At the end of the party, everyone realized that Emily had/hadn’t read the fable.
3. An island tour was scheduled in the afternoon, followed by a farewell dinner in the hotel lobby. All the tourists were very excited about it. Everyone assumed that Caroline would/wouldn’t taste the cocktail. It was soon obvious that Caroline had/hadn’t tasted the cocktail.

### Non-Denial Context

1. John enjoys having dinner with his parents but unfortunately he had to work late last week. His parents put the leftovers in the fridge. They wondered what John would do. The following morning his parents discovered that John had/hadn’t eaten the soup.
2. Emily was invited to surprise birthday party where the guests were asked to recite different literary fragments. Most of them chose less known authors. Everyone was curious what Emily would do. At the end of the party, everyone realized that Emily had/hadn’t read the fable.
3. An island tour was scheduled in the afternoon, followed by a farewell dinner in the hotel lobby. All the tourists were very excited about it. They didn’t know what Caroline would do. It was soon obvious that Caroline had/hadn’t tasted the cocktail.

## Appendix 3: *Illustrations of the iIems used in Experiment 6*

### Denial context

1. John enjoys having dinner with his parents but unfortunately he had to work late last week. His parents put the leftovers in the fridge. The following morning his parents discovered that, contrary to expectations, John had/hadn’t eaten the soup.

### Non-denial context

1. John enjoys having dinner with his parents but unfortunately he had to work late last week. His parents put the leftovers in the fridge. The following morning his parents discovered that John had/hadn’t eaten the soup.

## Footnotes

1 https://www.soscisurvey.de/

2 When determining the accuracy for each participant we took all the experimental sentences into account as well as half of the filler sentences, namely the negative filler sentences in the denial context and the affirmative filler sentences in the non-denial contexts. The other half of the filler sentences were not included in the calculation of accuracy because they could be answered with both ‘yes’ and ‘no’, depending how participants interpreted the task. For instance, a sentence like *Mark hasn’t fed the shelves* (negative non-denial filler) could receive the answer ‘yes’, standing for ‘yes, the sentence makes sense’ because people usually don’t feed shelves, and ‘no’, standing for ‘no, the sentence does not make sense’, as not feeding a shelf does not express a plausible event. The same holds for a sentence such as *Contrary to expectations, Mark has fed the shelves* for which the correct answer is ambiguous.

3 We also analyzed the data by means of repeated measures ANOVA with the factors *Polarity* (affirmative/ negative) and *Context* (denial/non-denial). The results revealed a main effect of Polarity (*F*(1,78) = 22.14, *p* < .001), with shorter RTs in the affirmative condition, a main effect of Context (*F*(1,78) = 145.1, *p* < .001), with shorter RTs in the non-denial contexts, but no interaction (*F* < 1).

4 The data were also analyzed by means of repeated measures ANOVA which revealed a main effect of Polarity (*F*(1,61) = 21.02, *p* < *.*001) and a main effect of Context (*F*(1,61) = 21.41, *p* < .001) but no polarity-by-context interaction (*F* < 1).

5 Additionally, we performed repeated measures ANOVA which revealed the same pattern of results as in the previous experiments: a main effect of *Polarity* (*F*(1,58) = 56.31, *p* < .001) and a main effect of *Context* (*F*(1,58) = 14.27, *p* < .001), but no significant interaction (*F* < 1).

6 The data were also analyzed by means of repeated measures ANOVA with the factors *Polarity* and *Context*. The results revealed a main effect of Polarity (*F*(1,63) = 28.58, *p* < .001) and a main effect of Context (*F*(1,63) = 22.61, *p* < .001) but the relevant interaction was not significant (*F*(1,63) = 0.06, *p* = .802).

7 Grice (1989) suggested four conversational maxims (Maxim of Quality, Quantity, Relation and Manner) based on a cooperative principle. Informativity and relevance refer to the Maxim of Quantity (“1. Make your contribution as informative as is required for the current purpose of the exchange; 2. Do not make your contribution more informative than is required”) and to the Maxim of Relation (“Be relevant”), respectively.

8 Repeated measures ANOVA were also carried out and the analysis revealed a main effect of polarity (*F*(1,66) = 18.92, *p* < .001, a main effect of context (*F*(1,66) = 177.2,* p* < .001) and a significant interaction between the factors *polarity* and *context* (*F*(1,66) = 19.05, *p* < .001).

9 Repeated measures ANOVA were also carried out and the analysis revealed a main effect of polarity (*F*(1,67) = 18.88, *p* < .001, a main effect of context (*F*(1,67) = 10.08,* p* < .001) and a significant interaction between the two factors (*F*(1,67) = 15.85, *p* < .001).
